# Decision Trees for Predicting Mortality in Transcatheter Aortic Valve Implantation

**DOI:** 10.3390/bioengineering8020022

**Published:** 2021-02-09

**Authors:** Marco Mamprin, Jo M. Zelis, Pim A. L. Tonino, Sveta Zinger, Peter H. N. de With

**Affiliations:** 1 Department of Electrical Engineering, Eindhoven University of Technology, 5612 AJ Eindhoven, The Netherlands; s.zinger@tue.nl (S.Z.); p.h.n.de.with@tue.nl (P.H.N.d.W.); 2 Department of Cardiology, Catharina Hospital, 5623 EJ Eindhoven, The Netherlands; jo.zelis@catharinaziekenhuis.nl (J.M.Z.); pim.tonino@catharinaziekenhuis.nl (P.A.L.T.)

**Keywords:** aortic valve disease, machine learning, one-year mortality prediction, outcome prediction, prognosis, transcatheter aortic valve implantation, TAVI

## Abstract

Current prognostic risk scores in cardiac surgery do not benefit yet from machine learning (ML). This research aims to create a machine learning model to predict one-year mortality of a patient after transcatheter aortic valve implantation (TAVI). We adopt a modern gradient boosting on decision trees classifier (GBDTs), specifically designed for categorical features. In combination with a recent technique for model interpretations, we developed a feature analysis and selection stage, enabling the identification of the most important features for the prediction. We base our prediction model on the most relevant features, after interpreting and discussing the feature analysis results with clinical experts. We validated our model on 270 consecutive TAVI cases, reaching a C-statistic of 0.83 with CI [0.82, 0.84]. The model has achieved a positive predictive value ranging from 57% to 64%, suggesting that the patient selection made by the heart team of professionals can be further improved by taking into consideration the clinical data we identified as important and by exploiting ML approaches in the development of clinical risk scores. Our approach has shown promising predictive potential also with respect to widespread prognostic risk scores, such as logistic European system for cardiac operative risk evaluation (EuroSCORE II) and the society of thoracic surgeons (STS) risk score, which are broadly adopted by cardiologists worldwide.

## 1. Introduction

**Figure 1:**
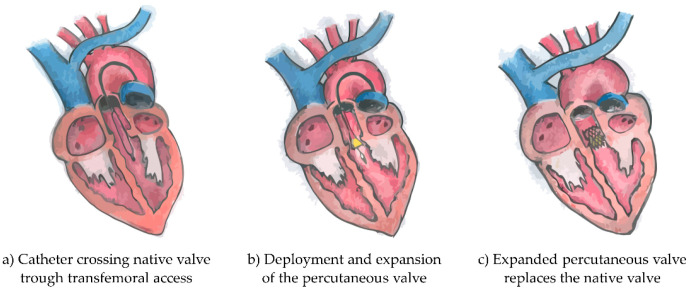
Diagrams of the three phases of transcatheter aortic valve implantation intervention. Originally the catheter crosses the native valve trough transfemoral access (**a**), follows the deployment and expansion of the percutaneous valve (**b**) and finally the expanded percutaneous valve replaces the native valve (**c**).

Degenerative aortic valve stenosis (AS) is the most common valvular heart disease in the developed world. If left untreated the disease has a devastating course, rapidly causing death when symptoms develop. AS is caused by calcification of the aortic valve (AV). This could also lead to aortic valve regurgitation (AR) which also causes heart failure. The treatment for severe aortic valve disease consisted until recently of surgical aortic valve replacement (SAVR). However, in recent years transcatheter aortic valve implantation (TAVI, diagram in Figure 1) has been developed and approved for use in severe- to intermediate-risk AV disease. Recently, two randomized controlled trials have been published where use in low-risk TAVI patients was non-inferior compared to SAVR. Despite the increasing development of this technique, there still is a risk bound to it. The broad use of TAVI in the last few years has shown a high chance of successful outcomes. However, the frailest patients sometimes do not benefit and can have complications after the procedure. The cause for this partial success is still not known.

Hence, careful patient selection is paramount. Identifying those patients who have improvements or those who are at a higher risk after TAVI is essential to maximize their survival, by providing an alternative treatment or therapy. Moreover, this would lead to an improvement in the use of the limited resources, which reduces the waiting lists. However, the identification of the patients that can have improvements from the TAVI procedure is a complex and still unsolved task because it is difficult to objectively quantify the improvements of a patient in a daily routine. Furthermore, the patients that are at a higher risk are often frail patients with several comorbidities and with an important medical history. Unfortunately, they can have severe complications, leading in the worst case to mortality, which further increases the motivation to find out why the identification was incorrect [1]. EuroSCORE II [2] and STS [3] risk scores are currently used for patient selection, assessing mortality risk at 30 days, but they were not developed for TAVI. Current risk scores or predictors specifically designed for assessing one-year mortality for TAVI are TAVI_2_-SCORe [4] and Arnold SV et al. [5]. Other predictors are also available but are specifically designed to predict 30 days mortality such as Iung et al. [6], Capodanno et al. [7], and Martin et al. [8]. Although several options are available, in current clinical practice EuroSCORE II and STS are still preferred because they are more extensively accepted as risk scores by the cardiologist worldwide and they consequently largely benefited from extensive validations.

With this paper, we aim to develop with a supervised machine learning approach, a very specific model to predict one-year mortality for the TAVI use-case. In fact, mortality at one year has been identified by the medical experts as the life expectancy threshold, above which the TAVI procedure is enabled and worth being performed. We aim to validate its performance on a dataset of 270 patients that have undergone TAVI in 2015 and 2016. All these patients were already selected by the heart team of clinicians, therefore, the decision whether to undertake TAVI or not was already made. The aim of this research is to develop a model that will have to be intended as a supporting tool available for consultation to provide further insights, whether required, to the current patient selection workflow. The heart team will always retain the role and responsibility for the final decision, thus assessing whether, or not, a patient will be an optimal candidate for TAVI.

We obtained promising prediction results and our work has the following contributions. Firstly, to the best of our knowledge, this is the first successful application of gradient boosting on decision trees for one-year mortality risk prediction of TAVI. Secondly, by applying a state-of-the-art model explanation technique, we have created useful feature insight that allows the medical doctors and us to interpret the model and perform an optimal feature selection. Thirdly, we have validated our model on a retrospective clinical dataset and we have compared our prediction results with other studies in literature and existing patient outcome predictors currently used in cardiac surgery.

## 2. Materials and Methods

### 2.1. Dataset

The anonymized dataset obtained from the Catharina Hospital consisted of 270 consecutive TAVI procedures that were performed between January 2015 and December 2016 using the following different brands and models of artificial aortic valves:

- SAPIEN XT (9 patients) and SAPIEN 3 (127 patients), Edwards LifeSciences (Irvine, CA, USA);
- CoreValve (10 patients) and CoreValve Evolut (124 patients), Medtronic (Minneapolis, MN, USA).

For most of the patients, the procedures were performed through standard transfemoral access and only 7.4% of them required an alternative access site, namely the subclavian artery (five patients), the transapical (seven patients), and the direct transaortic (eight patients).

The average age of the patients, when the TAVI procedure was performed, was 80.7 years, with a minimum age of 50.3 years and a maximum age of 94 years (standard deviation of 6.2 years). In our population, 48% of the patients were female.

**Figure 2:**
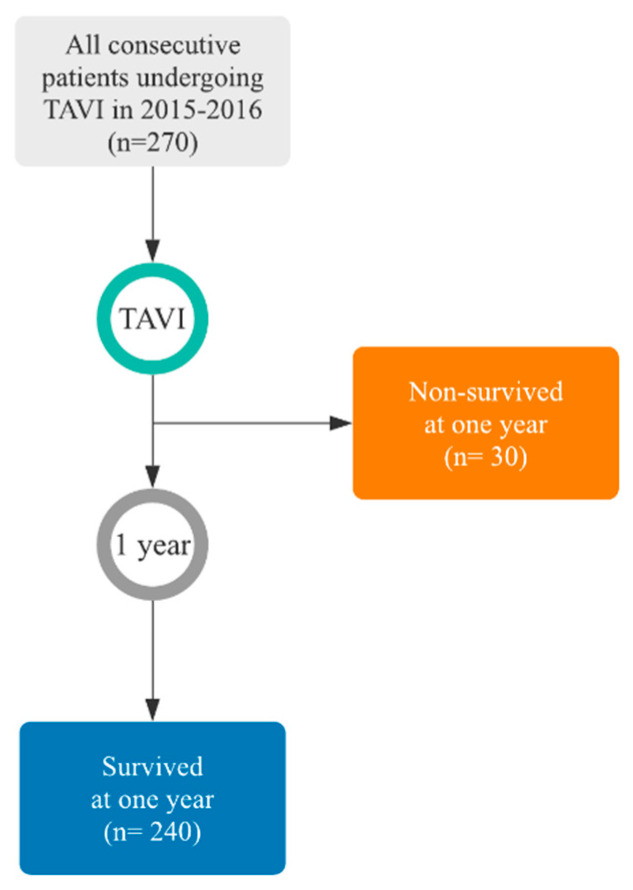
Flow of participants representing the study population. Survival distribution details are shown in Figure A2 (Appendix A).

The retrospective data were divided into two categories and one classification label was assigned to each category to create two different groups of patients according to their survival to one year from the date of TAVI, as shown in Figure 2. Therefore, we obtained the following two classes and groups:

- Survived within the first year (240 patients);
- Non-survived within the first year (30 patients, 50% of which did not survive within the first two months or did not survive to the procedure (five patients)).

The mortality information was collected in 2019 from the census of the national population of the Netherlands. The dataset consisted of numerous numerical and categorical information data, which could be divided, as shown in Table 1, in five principal categories: medical history, clinical data, patient questionnaires, risk scores, and medications. All clinical data were collected in the days and weeks prior to the TAVI procedure.

The retrospective study was approved by the local ethics committee and all enrolled patients signed informed consent. The reporting of this paper adheres to the “transparent reporting of a multivariable prediction model for individual prognosis or diagnosis” (TRIPOD) guidelines [6]. TRIPOD statement can be found in the supplementary materials.

### 2.2. Initial Processing of the Dataset

**Figure 3:**
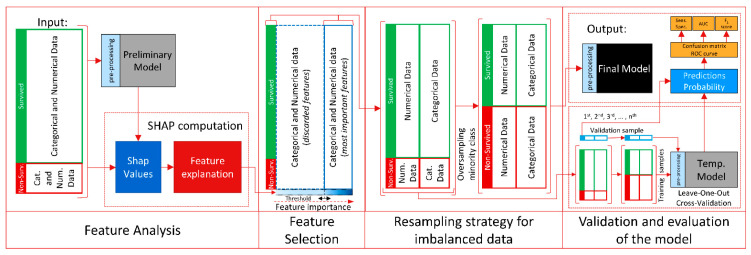
Diagram of the overall processing steps of the prediction model.

The overall processing steps of our data are depicted in Figure 3. The initial processing described here is preceding the diagram and is based on discriminating between numerical and categorical features. Once each feature is defined, we analyze each individual feature in detail, by considering the mean, range, and standard deviation of the numerical features, and the recurrence and instances of the categorical features.

We have reorganized some initially given features and some have been introduced, resulting from combining original features. For example, we mention the body mass index (BMI) computed from the height and the weight. An alternative feature processing has been applied for multiple answer questionnaires by using one-hot encoding. An additional approach has been reserved for the list of prescribed medicines. After analysis with the medical experts, we added and divided them into 28 pharmacological macro-categories (angiotensin-converting-enzyme inhibitors, alfa blocker, angiotensin II receptor antagonist, antiarrhythmic, antibiotics, anticoagulant, antidepressants, antiepileptic, anti-inflammatory, antiplatelet, benzodiazepines, beta-blocker, beta-2 sympathomimetics, calcium channel blockers, corticosteroids, diabetic medications, diuretics, hormones, inhalers, insulin, laxatives, nitrates, proton-pump inhibitors, renin inhibitors, statins, supplements, thyroid-stimulating hormone, others), according to their class of medication therapy and to their mechanism of functioning. Concerning the dates included in the original dataset, that were describing when certain events occurred prior to TAVI, it was decided to define them relative to the TAVI procedure date. Finally, we computed the intermediate and final scores (emotional well-being, energy/fatigue, general health, pain, physical functioning, role limitations due to emotional problems, role limitations due to physical health, social functioning) for the RAND-36 questionnaire [7] according to the clinical guidelines.

### 2.3. Gradient Boosting on Decision Tree Classifier

Gradient boosting on decision trees (GBDTs) is a supervised machine learning technique, which currently represents one of the state-of-the-art techniques for models based on decision trees. On the other hand, well-established neural network techniques have emerged in several fields including the one for cardiovascular outcome predictions, often providing promising results, with respect to other more classical machine learning techniques, when large datasets are involved [8,9,10]. Generally, decision trees are less data demanding and GBDTs techniques are typically optimal for small datasets, whereas neural networks usually perform better on large datasets [11]. In other words, decision trees can allow the model to reach optimal convergence without requiring those large datasets which are necessary for neural networks. Furthermore, decision trees offer better interpretability potential and, with respect to neural networks, they are better capable to handle categorical features (which are not differentiable by definition) in their decisional node splitting, while still accounting, as neural networks, for non-linear relationships. What has been mentioned goes in support of our choice to prefer, as classifiers for this study, decision trees over neural networks.

Recently, a new categorical feature-specific classifier called CatBoost has been designed that outperforms several gradient boosting on decision trees (GBDTs) classifiers [12,13] such as XGBoost [14] which does not have a dedicated pre-processing for categorical features and LightGBM [15] which is not advisable on small-scale data.

The CatBoost classifier was used, since in clinical practice and consequently in this dataset, categorical features are common. Therefore, we can more easily exploit all the information provided in the dataset, leaving this innovative approach for pre-processing of the categorical and numerical features to the preliminary stage of CatBoost (see Figure 3), at each input stage of each model. In fact, at the preliminary stage, CatBoost converts all the categorical features into numerical data by incorporating the recurrence of each instance, missing values are considered as an instance of the feature. Numerical features are then processed by aggregating different feature values in a histogram, which is specifically optimized for an efficient and fast memory access and elaboration. Missing values are processed as the minimum value of that feature to guarantee the split in the decision tree and to separate them more effectively from other numerical features.

The second stage of CatBoost involves an algorithm that builds an ensemble model with an iterative approach. At the 1st iteration, the algorithm learns from the dataset the first decision tree, to reduce the training error. At the 2nd iteration, the algorithm learns from the dataset one more decision tree, to reduce the error made by the decision tree obtained at the 1st iteration and the algorithm repeats this procedure for all succeeding iterations until the iteration count is exceeded. This count is chosen to maximize the training of the model without overfitting the data and, therefore, reducing the generalization capabilities of the model.

### 2.4. Feature Analysis and Selection

The feature analysis stage is achieved by exploiting a new method that, with a local-level approach and its foundations in game theory, is able to provide interpretations and explanations of machine learning models, as shown in the first stage of Figure 3.

We exploited the SHapley Additive exPlanations unified approach [16,17], using Shapley values, which is a technique recently published and applied to compute the importance of each feature of a model. Once extracted, using all the importance values for each feature, we compute the following average:

(1) $$\varphi_{m}=\frac{\sum_{j=1}^{n}\left|\phi_{j,m}\right|}{n}$$

where $\phi_{j,m}$ is the importance value of the feature *m* for a patient *j* on the total amount of *n* patients. We obtain then the estimate of the most important features in the decision making of the model. Consequently, we reorder them from the most relevant downwards by reordering the feature vector list.

The most important features were then discussed with the medical experts to find possible patterns and clinical explanations related to patient mortality. The validated relevant features which were confirmed by the clinical experts and that were found more discriminative in the decision process were used to train the final model.

### 2.5. Classifier Training and Resampling Strategy

At the feature analysis stage, we trained the classifier on a deep decision tree by including the entire dataset. We then analyzed the model to evaluate the importance of each feature. The temporary and the final model were trained only with the most informative features by imposing a threshold, to discard all non-relevant features. We conducted also a visual inspection of the SHapley Additive exPlanations summary plot, which proved to be useful when discussing the results with the medical experts.

The training parameters of the final model were chosen after performing hyperparameter research, further details are shown in Table 2. Different combinations of possible parameters have been iterated as well as different levels of feature thresholds, ranging from the most important two to the most important 40.

We then identified which were more suitable for maximizing the F1 score and AUC metric, by performing *k*-fold cross-validation multiple times with different randomizations per iteration. The temporary models used for validation utilize the same parameters as the final one.

The intrinsic imbalanced nature of the medical datasets, as known in the literature [18], led us to apply a random over-sampling strategy to the minority class (non-survived patients) until a balanced ratio with the majority class (survived patients) was reached.

### 2.6. Validation and Evaluation

Validation of the model was been performed with five-times leave-one-out cross-validation (LOOCV), with a dedicated pipeline for each iteration. In fact, the previously discussed random over-sampling of the minority class is applied at each iteration, but only after each test sample is removed, to reduce any possible uncertainty in the results that could be caused by the randomization of the minority class samples. LOOCV was chosen because the minority class population was limited to only 30 patients and consequently a sufficient amount of information would not have been available to train the model if a more sophisticated validation approach would have been implemented (i.e., training, test, and validation set split). Current state-of-the-art prognostic risk scores in cardiac surgery use the area under the curve (AUC) of the receiver operating characteristic curve (ROC or C-statistic) as the benchmark metric. However, this metric is not always the best choice, especially in the case of validation performed on a dataset with imbalanced classes. Therefore, we adopted other metrics such as sensitivity, specificity, accuracy, precision (or positive predictive value), and F1 score. As suggested in the literature, calibrated risk models are of vital importance for valid decision support [19,20]. Therefore, we reported also a calibration study for each of the classifiers that have been implemented. Due to the small size of the dataset, we did not train any regression model to calibrate the classifiers.

## 3. Results

### 3.1. Selected Features

According to the results obtained with the SHapley Additive exPlanations and the related discussion of these results with the cardiologists, we identified the most relevant features for the prediction of one-year mortality for TAVI.

**Figure 4:**
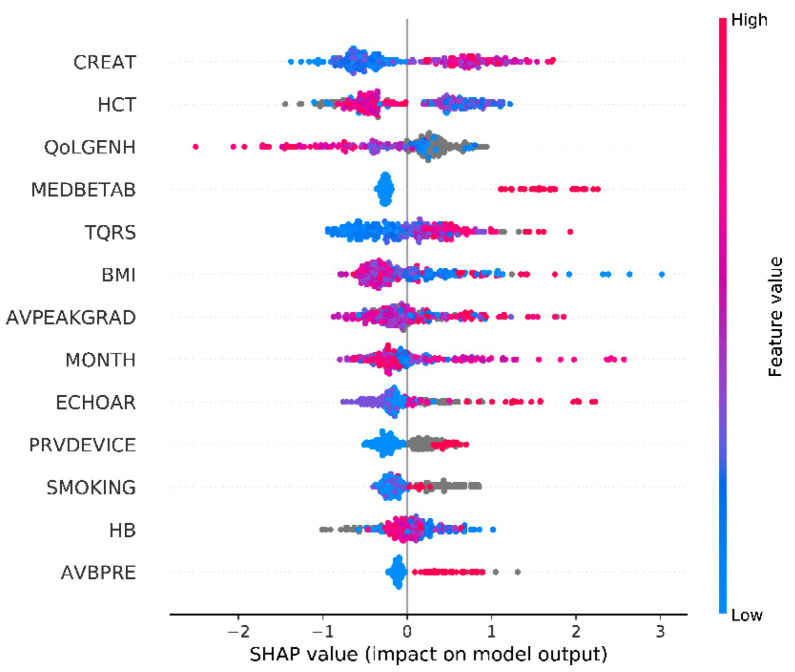
SHapley Additive exPlanations summary plot (missing values are shown in grey).

The selected features are presented in alphabetical order and in order of importance, according to the SHapley Additive exPlanations, in Table 3 and in Figure 4, respectively.

Other relevant features that have been discarded to maximize the results at the risk of a reduced generalization capability on another population are risk scores related to bleeding with antiplatelet and nonsteroidal anti-inflammatory drugs (NSAIDs), New York heart association functional classification (NYHA class), QTC interval, gender, glomerular filtration rate (GFR), recent coronary artery bypass grafting (CABG) interventions, previous myocardial infarction and further classes of medicines e.g., angiotensin-converting-enzyme (ACE) inhibitors, angiotensin II receptor blockers (ARBs), and proton pump inhibitors (PPIs).

We emphasize here that several factors are influencing the feature selection and the classifier training. Particularly, the order of the most important features can be highly influenced by the missing values. In fact, a certain feature can ascend or descend the list of the most important features proportionally to the amount of its missing values. It must be said that missing values contain information as well because they are a natural consequence of the clinical workflow, of the internal protocols adopted by each hospital, and of the varying urgencies that characterize each patient journey. However, by including all the features shown in Table 3 in the final model, we ensured an optimal overview of the patient, acquired with a multi-disciplinary approach, which has shown promising generalization capability on this dataset.

### 3.2. Evaluation of the Model

In Table 6, the performance of the mortality predictor achieved in this work is compared to the ones achieved in other studies available in the literature. As previously mentioned, EuroSCORE II and STS, which are extensively accepted as risk scores by the cardiologist worldwide, reached in a TAVI study AUCs of 0.81 and 0.77, respectively. However, only 59 symptomatic patients with severe aortic stenosis were selected for this 30-day mortality study [21]. Two prognostic risk scores for one-year mortality prediction for the TAVI procedure are the TAVI2-SCORe [4] which when validated on 511 patients, showed an AUC of 0.72, and the Arnold SV et al. [5] which when validated on 1830 patients, achieved an AUC of 0.66. Other 30-day mortality predictors for TAVI which are worth mentioning for comparison as they have been validated on a large population, are the ones presented in the works of Iung et al. [22], Capodanno et al. [23], and Martin et al. [24]. On population sizes of 3833, 1878, and 6339, they reached AUCs of 0.59, 0.71, and 0.66, respectively.

**Figure 5:**
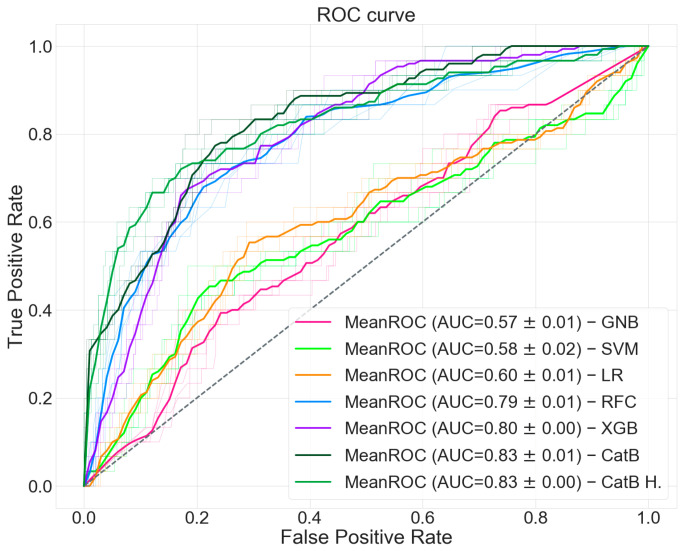
ROC curves of the classifiers.

We computed the ROC curve shown in Figure 5 with an AUC of 0.83. We reached an accuracy of 0.90 with a specificity of 0.97 and a sensitivity of 0.37. Finally, we computed an F_1_ score of 0.45. All the evaluation metrics and classification predictions acquired during the validation of our prediction model are shown in Table 4 as value ± standard deviation and Table 5 as computed patient amounts. As a comparison, we re-performed the entire validation pipeline with other well-known machine learning classifiers. For this purpose, numerical values were scaled in the range ±1, replacing missing values with zero and one-hot encoding was applied for categorical features, considering missing values as an instance apart. Numerical missing values have been replaced also with the mean, weighted mean, median, weighted median, and minimum value of the numerical feature, leading to slightly worse results.

**Figure 6:**
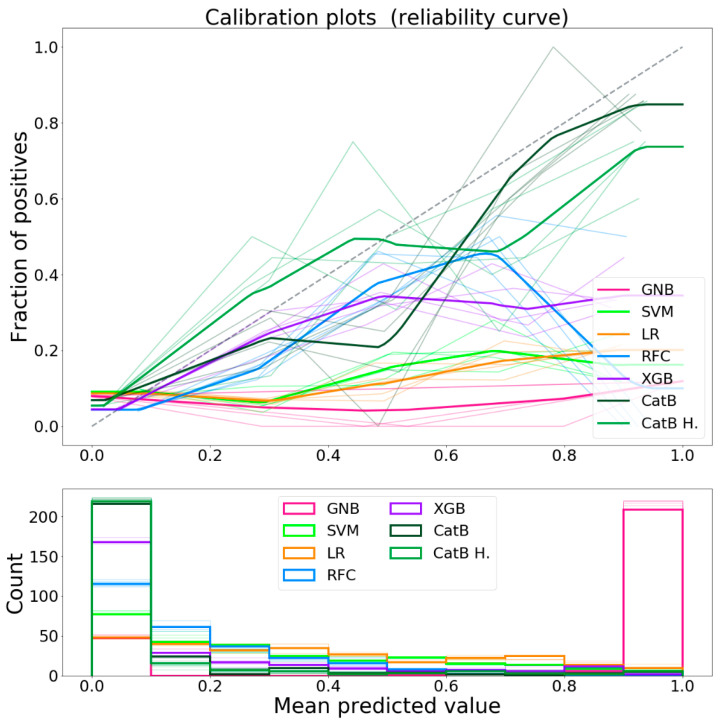
Calibration curves of the classifiers.

The calibration curves for each classifier are shown in Figure 6. As previously mentioned, the classifiers calibration was not been performed because the data available was not sufficient to generate a regression model to calibrate the output. Despite the uncalibrated predictions, CatBoost was shown to have, with respect to the other classifiers, a calibration curve that approaches the ideal one.

As shown in Table 5, our prediction model was able to identify from 10.0 to 11.0 patients (true positive) that would not benefit from TAVI. An amount that grows from 5.8 to 8.2 patients being misclassified (false positive) suggesting these patients would not benefit from TAVI, although being good candidates. Despite the small number of false positives, a positive predictive value (precision) ranging from 57% to 64% is achieved, suggesting that improvements of the patient selection with respect to the current clinical workflow is possible. In this regard, the other classifiers have shown not comparable prediction capabilities.

As shown in Table 6, the prediction model presented in this study has shown promising potential to evaluate the risk score for TAVI patients, with respect also to current state-of-the-art or other studies predictors. It should be noted that one-year mortality is generally more challenging than 30-day mortality or in-hospital mortality.

## 4. Discussion

To clarify and discuss the findings of SHapley Additive exPlanations, clinical experts were consulted to better understand why certain features were considered important for the prediction and for an improved understanding of the possible interaction between all the different variables. With the goal of finding an answer to why certain features were considered more important than others in the decision making of the model, we infer the following conclusions.

Severe AV regurgitation is linked to heart failure and mortality, which is found also in our analysis. Paravalvular regurgitation after TAVI is a common complication. Paravalvular regurgitation is most common in patients with severe calcification of the native aortic valve leaflets. When a TAVI valve is deployed, it may not be able to fully operate properly, resulting in leakage. The severity of the regurgitation is correlated to a worse prognosis since patients will develop heart failure and require in-hospital treatment.

A low AV peak gradient can be related to less severe aortic stenosis or to a dysfunctional left ventricle. High values indicate severe/critical aortic stenosis, that if left untreated, can lead to mortality for sudden cardiac death or heart failure. In severe aortic valve stenosis, the valve is narrowed resulting in a higher resistance over the aortic valve. The left ventricle needs to develop a higher pressure to open the valve. Severe symptomatic aortic valve stenosis is a devastating disease if left untreated and has a high mortality rate.

Atrioventricular block is linked to higher syncope and mortality and needs to be treated with a pacemaker giving complications, which reduces survival. Atrioventricular block is a common complication after TAVI. This complication is related to valve size and calcifications of the native aortic valve. The atrioventricular node is positioned in the septum of the ventricle and when a TAVI valve is deployed, the stent frame or calcifications of the native valve can impose pressure on the node causing damage, which in turn results in several degrees of atrioventricular block. If left untreated, this can cause syncope or even heart failure. Monitoring the first days after TAVI for the development of atrioventricular blocks is required, as the function may sometimes return. If not, a pacemaker is implanted to bypass the node.

Beta-blocker class of medication is used to manage heart rhythms (atrial fibrillation or ventricular arrhythmia) and in the case of heart failure. Both cases have an impact on survival. Atrial fibrillation is often seen in patients with aortic valve stenosis. To treat this rhythm problem, beta-blockers are often the first choice for rate control.

Extreme values of body mass index (BMI) are unhealthy factors, even though there is clinical relevance that being slightly overweight gives a better prognosis after surgery.

High blood creatinine concentrations suggest that there is a kidney deficiency. Kidney failure is linked to higher mortality. A high blood creatinine concentration is an indication that the renal function is declined.

Very low values for the RAND-36 [27] general health score indicate that the life quality of the patient is very low. This can be related to other comorbidities that reduce survival.

A low hematocrit and hemoglobin value may suggest anemia, which is linked to mortality. Patients with anemia may require a higher cardiac output to deliver oxygen to the organs. Patients with aortic stenosis can develop heart failure and are thus unable to increase their cardiac output sufficiently. This may result in the deterioration of patients’ hemodynamics and eventually could increase mortality.

**Figure A3:**
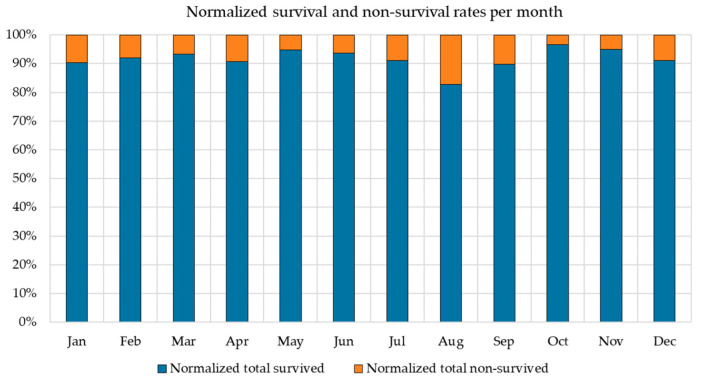
Survival and non-survival rates per month.

The month on which each patient faces its post-procedure recovery has been shown to be statistically related to survival. Our assumption has been verified on the survival distribution of another center showing a similar survival trend over certain months, as shown in Figure A3 (Appendix A). Certain cardiovascular complications are temperature-related, while seasonal diseases also impact survival. This has to be more carefully considered as part of the comorbidities affecting the post-procedure recovery.

Previous devices such as pacemakers are implanted when rhythm disturbances are present and may lead to long-term complications. Implantable cardioverter-defibrillator (ICD) or cardiac resynchronization therapy with defibrillator (CRT-D) are implanted when the left ventricular function is very low, or when the patient has deadly arrhythmia. Both factors lead to a lower life expectancy.

A high QRS duration is an index of a more diseased conduction system. This can lead to heart failure. The conduction system can be damaged by TAVI valves. As the valve is deployed, the stent frame or the calcification of the aortic valve may cause temporary or permanent damage to the conduction system. This can lead to a further widening of the QRS on the electrocardiogram. In some studies, this is linked to a higher mortality.

Smoking leads to a higher risk of any cardiovascular disease, again proof of the fact that smoking leads to a lower chance of survival after an operation.

This twofold approach, technical and clinical, provides a more solid background for this research, enabling the identification of important features for the prediction incorporating clinical expertise and the capability of analyzing a large amount of clinical data exploiting extensive and computationally intensive feature analysis.

## 5. Limitations of the Study

Similar conclusions of the most important features that have been identified cannot be guaranteed on a larger population or on another clinical center, especially when considering datasets larger than one or two orders of magnitude in size. The validation approach used in this research was chosen because of the clear limitations resulting from a small dataset size. Furthermore, extending the validation on a larger dataset would assess the prediction potential of this model on a larger population, possibly using other validation techniques that take advantage of a threefold split for training, validation, and test set. As this research is a single-center study, we firmly believe that external validations would give added value to the research field. Cross validating the models which are available in the literature could provide validation metrics completely exempt from bias that might be present on each center dataset and could, therefore, lead the model to non-ideally fit or overfit the data. This can represent a risk to be considered when validations are performed on single-center studies, especially when small populations with imbalanced classes are involved.

## 6. Conclusions

With this research, we aim to develop a predictive model for patient selection in cardiac surgery, specifically for the TAVI use-case, by exploiting a new state-of-the-art machine learning classifier designed for categorical features. We have successfully identified the most important features to predict the one-year mortality and have developed a model based on the most relevant features. We validated our model on 270 patients, obtaining promising results also with respect to widespread prognostic risk scores or predictive models for TAVI. The model is able to discriminate the two populations of survived and non-survived patients with an AUC on the ROC of 0.83 with CI [0.82, 0.84] and with a specificity and sensitivity of 0.97 with CI [0.96, 0.97] and 0.37 with CI [0.28, 0.45], respectively. Since our dataset was intrinsically imbalanced with a ratio of 1/8, we measured also the F_1_ score as an evaluation metric for this model, which resulted in a value of 0.45 with CI [0.36, 0.53]. The model has achieved a positive predictive value (precision) ranging from 57% to 64%, suggesting that the patient selection with respect to the current clinical workflow can be improved. It is important to extend the validation of the model on other patient data, possibly by performing a validation on a larger dataset, as well as in an inter-center validation process. Summarizing, we evaluated the predictive potential of a model developed for exploiting ML techniques on a set of features found to be important for one-year survival. The model has shown promising predictive potential for the TAVI procedure, offering attractive and powerful insights and results for the prediction of one-year mortality. The methodology presented in this study can be easily scaled up incorporating larger datasets and can be translated to other use-cases. This preliminary study forms a solid basis for further technical investigation and clinical studies to further consider, validate, and extend our approach.

## Figures and Tables

**Table 1:** Complete dataset information.

Type	Name
**Medical History**	
Ischemic heart disease	History of ischemic heart disease, history of or current multi-vessel coronary artery disease, history of or current left main coronary artery stenosis, previous myocardial infarction (MI), single or multiple MI, date and location of most recent MI, previous percutaneous coronary intervention (PCI), single or multiple PCI, date and location of most recent PCI, previous coronary artery bypass grafting (CABG), single or multiple CABG, date of most recent CABG
Valvular heart disease	Previous valvular operation, previous aortic/mitral/pulmonic/tricuspid valve operation, actual valvular disease, aortic stenosis or regurgitation, mitral stenosis or regurgitation or valve prolapse, pulmonic stenosis or regurgitation, tricuspid stenosis or regurgitation, endocarditis
Chronic heart failure	Chronic heart failure, congestive heart failure (CHF) hospitalization, cardiomyopathy, cardiac transplant
Arrhythmias and conduction disorders	Sinus node dysfunction, supraventricular tachycardia, atrial fibrillation, atrial flutter
Previous devices	Previous devices such as a pacemaker (PM), or implantable cardioverter defibrillators (ICD), or cardiac resynchronization therapy (CRT), or implantable event recorder, date of last implantation, type of device
Risk factors	Hypertension, hypercholesterolemia, smoking, years as a smoker, smoking date of cessation, alcohol misuse, family history of sudden cardiac death, family history of vascular disease
Risk scores related factors	HAS-BLED Score: prior major bleeding/predisposition, HAS-BLED Score: labile INR, HAS-BLED Score: antiplatelets, NSAID
Other diseases	Cerebrovascular disease, cerebrovascular disease affecting activities of daily living, cerebrovascular surgery, poor mobility, diabetes, chronic lung disease, peripheral artery disease and surgery, pulmonary hypertension, porcelain aorta, chronic renal failure and eventual therapy, liver cirrhosis, deranged liver tests, malignancy
**Clinical Data**	
Functional class and clinical examination	Symptoms and date of the events, NYHA class, CCS class, height, weight, BMI
Laboratory examination	Date and location, hemoglobin, hematocrit, ASAT, LDH, CK, CK-MB, Creatinine, GFR(MDRD), INR, NT-pro-BNP
Rhythm	Date and location, heart rate, rhythm type, PR interval, QRS duration, QT interval (lead II), QTc interval, atrioventricular block, bundle branch block
Echocardiography	Date and location, left ventricle (LV) end-diastolic and end-systolic diameters, LV end-diastolic volume (biplane), LV quantitative and qualitative ejection fraction, right ventricle (RV) quantitative ejection fraction, heart frequency, LV outflow tract (LVOT) diameter, LVOT peak velocity, LVOT velocity–time integral, AV peak velocity, AV peak and mean gradient, aortic valve area, aortic and mitral and tricuspid valve regurgitation, maximal RV systolic velocity
**Risk Score**	
Risk score	Identification of seniors at risk—hospitalized patients (ISAR-HP) frailty score, Cockcroft–Gault clearance
**Patient Questionnaire**	
Medical information and problems	Symptoms and events, NYHA class, CCS class
Daily activities	Edmonton frailty questionnaire
Quality of life	RAND-36 quality-of-life questionnaire
**Medications**	
Medication	Prescribed medicines

**Table 2:** Hyperparameter setting.

Parameter	Iterated Values
Tree depth	5, 6, 8
Number of trees (iterations)	100, 200, 300, 400
Learning rate	0.15
One-hot encoding max size	2, 3, 4, 12
Evaluation metric	Precision, F1, AUC
Loss function	Log loss
L2 leaf regularization term	3, 6, 12
Validation	Stratified 10-fold cross-validation repeated five times

**Table 3:** Details of the most important features for the prediction. Numerical data distribution is shown in Figure A1 (Appendix A).

Description	Unit	Abbreviation	Mean Value ± SD or Instances (*)	
			Survived (240)	Non-Survived (30)
AV regurgitation	**-**	ECHOAR	No (83) Mild (90) Moderate (19) Severe (13)	No (12) Mild (6) Moderate (2) Severe (6)
AV peak gradient	mmHg	AVPEAKGRAD	75.21 ± 26.70 (171)	64.87 ± 37.01 (21)
Atrioventricular block	-	AVBPRE	No (200) AV-block (34) AV-block III (2)	No (18) AV-block (7) AV-block III (2)
Beta blockers class of medicines	-	MEDBETAB	Yes (26) No (214)	Yes (11) No (19)
Body mass index	kg/m^2^	BMI	26.93 ± 4.21 (239)	26.17 ± 4.93 (30)
Creatinine	μmol/L	CREAT	105.65 ± 50.14 (235)	122.43 ± 51.30 (30)
General health score	0–100	QoLGENH	37.67 ± 14.51 (104)	22.14 ± 13.50 (7)
Hematocrit	%	HCT	39 ± 5 (209)	36 ± 3 (30)
Hemoglobin	mmol/L	HB	7.89 ± 1.03 (205)	7.38 ± 0.81 (29)
Month of post-procedure recovery	-	MONTH	Jan, …, Dec (Figure A3 Appendix A)	Jan, …, Dec (Figure A3 Appendix A)
Previous devices	-	PRVDEVICE	No (129) Yes (16)	No (7) Yes (4)
QRS duration	msec	TQRS	109.67 ± 27.75 (234)	122.81 ± 34.01 (26)
Smoking status	-	SMOKING	No (121) Former (45) Actual (14)	No (8) Former (3) Actual (1)

* Population size in brackets.

**Table 4:** Results of the TAVI mortality prediction model and comparison with other classifiers.

Metrics	CatB H. ^1^	CatB ^2,^*	XGB ^3,^*	RFC ^4,^*	LR ^5,^*	SVM ^6,^*	GNB ^7,^*
Sensitivity	0.37 ± 0.06 [0.28, 0.45]	0.33 ± 0.02 [0.30, 0.36]	0.35 ± 0.04 [0.30, 0.41]	0.14 ± 0.05 [0.07, 0.21]	0.53 ± 0.01 [0.51, 0.55]	0.49 ± 0.02 [0.46, 0.51]	0.87 ± 0.00 [0.87, 0.87]
Specificity	0.97 ± 0.00 [0.96, 0.97]	0.98 ± 0.00 [0.97, 0.98]	0.91 ± 0.01 [0.90, 0.92]	0.97 ± 0.01 [0.96, 0.98]	0.72 ± 0.01 [0.70, 0.74]	0.74 ± 0.01 [0.73, 0.75]	0.20 ± 0.01 [0.18, 0.21]
Accuracy	0.90 ± 0.01 [0.89, 0.91]	0.90 ± 0.00 [0.90, 0.91]	0.85 ± 0.01 [0.84, 0.86]	0.88 ± 0.01 [0.87, 0.90]	0.70 ± 0.01 [0.68, 0.71]	0.71 ± 0.01 [0.70, 0.73]	0.27 ± 0.01 [0.26, 0.28]
Precision	0.57 ± 0.06 [0.49, 0.65]	0.64 ± 0.05 [0.57, 0.70]	0.33 ± 0.03 [0.28, 0.37]	0.40 ± 0.13 [0.23, 0.58]	0.19 ± 0.01 [0.18, 0.20]	0.19 ± 0.01 [0.18, 0.20]	0.12 ± 0.00 [0.12, 0.12]
F_1_ score	0.45 ± 0.06 [0.36, 0.53]	0.44 ± 0.02 [0.41, 0.47]	0.34 ± 0.03 [0.29, 0.39]	0.21 ± 0.07 [0.11, 0.30]	0.28 ± 0.01 [0.27, 0.29]	0.27 ± 0.01 [0.26, 0.29]	0.21 ± 0.00 [0.21, 0.21]
AUC-ROC	0.83 ± 0.00 [0.82, 0.84]	0.83 ± 0.01 [0.83, 0.84]	0.80 ± 0.00 [0.80, 0.81]	0.79 ± 0.01 [0.78, 0.80]	0.60 ± 0.01 [0.59, 0.61]	0.58 ± 0.02 [0.56, 0.60]	0.57 ± 0.01 [0.56, 0.59]

Results are shown as value ± standard deviation, within brackets is the confidence interval at 95%. * Default parameters were used, ^1^ CatBoost with hyperparameters, ^2^ CatBoost [12,13], ^3^ XGBoost [14], ^4^ random forest classifier [25], ^5^ logistic regression, ^6^ support vector machine [26], ^7^ Gaussian naïve Bayes.

**Table 5:** Classification predictions of the TAVI mortality prediction model and comparison with other classifiers.

Classification Predictions ^†^	CatB H. ^1^	CatB ^2,^*	XGB ^3,^*	RFC ^4,^*	LR ^5,^*	SVM ^6,^*	GNB ^7,^*
True positive	11.0 [8.5, 13.5]	10.0 [9.1, 10.9]	10.6 [8.9, 12.3]	4.2 [2.2, 6.2]	15.8 [15.2, 16.4]	14.6 [13.9, 15.3]	26.0 [26.0, 26.0]
False negative	19.0 [16.5, 21.5]	20.0 [19.1, 20.9]	19.4 [17.7, 21.1]	25.8 [23.8, 27.8]	14.2 [13.6, 14.8]	15.4 [14.7, 16.1]	4.0 [4.0, 4.0]
True negative	231.8 [230.2, 233.4]	234.2 [232.6 235.8]	218.4 [215.7, 221.1]	233.8 [231.6, 236.0]	172.8 [168.9, 176.7]	177.8 [174.6, 181.0]	47.0 [43.7, 50.3]
False positive	8.2 [6.6, 9.8]	5.8 [4.2, 7.4]	21.6 [18.9, 24.3]	6.2 [4.0, 8.4]	67.2 [63.3, 71.1]	62.2 [59.0, 65.4]	193.0 [189.7, 196.3]

^†^ Positive class is referred to the 30 patients that did not survive to the first year, values contain decimals because, after the five repetitions of the leave-one-out cross-validation, the confusion matrices are averaged. Within brackets are the confidence intervals at 95%. * Default parameters were used, ^1^ CatBoost with hyperparameters, ^2^ CatBoost [12,13], ^3^ XGBoost [14], ^4^ random forest classifier [25], ^5^ logistic regression, ^6^ support vector machine [26], ^7^ Gaussian naïve Bayes.

**Table 6:** Performance analysis with respect to other mortality predictors assessed on data from other centers and available in the literature.

Model Name	Our Approach	TAVI2 SCORe ^1^	Arnold SV et al. ^2^	Euro SCORE II ^3^	STS Risk Score ^4^	Iung et al. ^5^	Capodanno et al. ^6^	Martin et al. ^7^
AUC-ROC	0.83	0.72	0.66	0.81	0.77	0.59	0.71	0.66
Population	270	511	2830	59	59	3833	1878	6339
Mortality	one-year	one-year	one-year	30-day	30-day	30-day	30-day	30-day

^1^ TAVI2-SCORe [4], ^2^ Arnold SV et al. [5], ^3^ EuroSCORE II [21], ^4^ STS Risk Score [21], Iung et al. (France TAVI registry) ^5^ [22], Capodanno et al. (OBSERVANT score, Italy TAVI registry) ^6^ [23], Martin et al. (UK-TAVI Registry) ^7^ [24].

**Figure A1:**
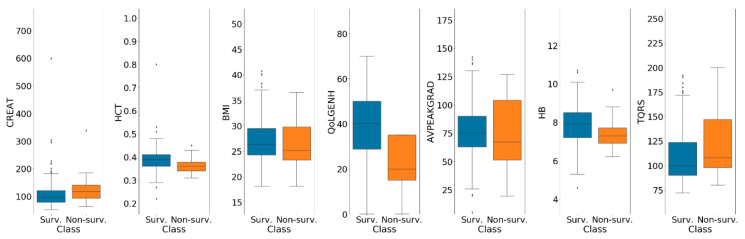
Boxplots of numerical features are shown for both classes of the population. Non-survived and survived patient distributions are shown in orange and blue, respectively.

**Figure A2:**
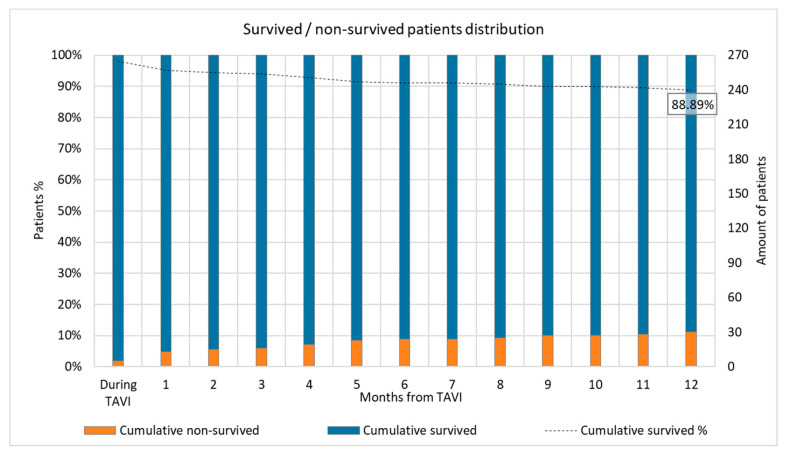
Survival distribution over the first year.

## Data Availability

The data presented in this study are not publicly available due to privacy and ethical restrictions. Data were obtained from Catharina Hospital (Eindhoven, the Netherlands) and have been made available after the permission and approval, through formal request, of the Catharina Hospital ethical committee. The models presented in this study are available for further validation and cross-validation studies with other centers. Contact the corresponding author to this extent.

## Supplementary Materials

The TRIPOD statement is available online at https://www.mdpi.com/2306-5354/8/2/22/s1.

## Appendix A

### Data Distribution

Boxplots representing the numerical features distribution for both classes of the population are shown in Figure A1.

Further details concerning survival distribution over the first year of the population are shown in Figure A2.

In Figure A3, information about survival for two TAVI centers for a total amount of 2183 patients is shown.
